# Physiochemical Modeling of Vesicle Dynamics upon Osmotic Upshift

**DOI:** 10.1016/j.bpj.2019.11.3383

**Published:** 2019-12-14

**Authors:** Matteo Gabba, Bert Poolman

**Affiliations:** 1 Department of Biochemistry, Groningen Biomolecular Sciences and Biotechnology Institute, University of Groningen, Groningen, the Netherlands

## Abstract

We modeled the relaxation dynamics of (lipid) vesicles upon osmotic upshift, taking into account volume variation, chemical reaction kinetics, and passive transport across the membrane. We focused on the relaxation kinetics upon addition of impermeable osmolytes such as KCl and membrane-permeable solutes such as weak acids. We studied the effect of the most relevant physical parameters on the dynamic behavior of the system, as well as on the relaxation rates. We observe that 1) the dynamic complexity of the relaxation kinetics depends on the number of permeable species; 2) the permeability coefficients (P) and the weak acid strength (*pK*_*a*_-values) determine the dynamic behavior of the system; 3) the vesicle size does not affect the dynamics, but only the relaxation rates of the system; and 4) heterogeneities in the vesicle size provoke stretching of the relaxation curves. The model was successfully benchmarked for determining permeability coefficients by fitting of our experimental relaxation curves and by comparison of the data with literature values (in this issue of *Biophysical Journal*). To describe the dynamics of yeast cells upon osmotic upshift, we extended the model to account for turgor pressure and nonosmotic volume.

## Significance

Physiochemical kinetic models are an important set of tools for the understanding of biochemical and biological systems. We present a comprehensive description of the relaxation dynamics of vesicles upon osmotic shock that includes volume variation, reaction kinetics, passive permeability across the membrane, and turgor pressure. The model is a flexible platform for the description of many biochemical systems. Owing to its generality, the model is easily extended with the addition of new features for the interpretation of in vitro experiments with protein transporters, electrochemical studies, and uptake experiments.

## Introduction

Out-of-equilibrium (pump-and-probe) relaxation techniques, which are methods in which the relaxation kinetics of the system are measured upon perturbation of the equilibrium, are precious experimental tools to investigate the behavior and properties of many systems in the life, chemical, and physical sciences. Indeed, the measured relaxation kinetics contains invaluable information about the microscopic properties of such systems. Despite being relatively easy to perform, traditional pump-and-probe experiments crucially rely on modeling of the system’s dynamics for correct data interpretation. In this respect, chemical kinetics and physiochemical dynamic models are widely used. Perfect examples are osmotic-shock relaxation experiments performed on lipid vesicles to characterize the membrane physiochemical properties (1, 2, 3). In this issue of *Biophysical Journal* (4), we present a stopped-flow fluorescence-based assay for measurement of weak acid or base permeation across the membrane of both artificial vesicles and living cells. Despite the long-term application of osmotic-shock relaxation techniques, we could not find in the literature any satisfactory representation of our experimental systems. Thus, we set out to construct a comprehensive theoretical model describing the vesicle dynamics upon osmotic perturbation. The model was benchmarked with the osmotic-shock relaxation data (4) and allowed us to obtain permeability coefficients of both weak acids and water. Then, we modified the model to describe yeast cell dynamics upon osmotic shock and determine the permeability coefficients of weak acids across the plasma membrane. Importantly, the model is a flexible platform for the description of many biochemical systems. Owing to its generality, the model is easily extended and upgraded with the addition of new features—for instance, in vitro experiments with protein transporters and electrochemical studies—but it can also be used to characterize in vivo uptake experiments.

## Theory

We describe vesicle relaxation upon osmotic shock to understand the relationship between the microscopic properties and the dynamics of the system. A general framework for the solution of this problem with constant vesicle volume is given by Knudsen (5). We aim to extend the description to account for volume variations. We focus on passive diffusion either across the lipid bilayer or through protein channels.

### Vesicle relaxation dynamics

#### Problem definition

We describe a spherical vesicle of volume *V* delimited by a flexible membrane with constant surface area *A*. We assume that the membrane thickness *d* is much smaller than the vesicle radius *r*_0_. The vesicle entraps *n* molecular species and freely diffuses in a solution containing *m* species. All molecular species are permeable across the membrane. We assume that the solute diffusion in solution is much faster than the diffusion across the physical barrier, that is, the vesicle membrane, which is the limiting step for the relaxation dynamics on timescales longer than milliseconds. Thus, the solutions are well mixed, and both the internal *c*_*i*_ and external $c_{i}^{*}$ concentrations are spatially uniform, that is, *c*(*r*) = *c*. Also, because we assume that the external-phase volume is much larger than the vesicle volume *V*, the external concentrations $c_{i}^{*}$ are constant in time. Finally, we assume that the internal and external solutions are electrically neutral and that charged molecules are impermeable across the membrane. To simplify the text, we omit the temporal dependences of the following quantities: *c*_*i*_(*t*), *N*_*i*_(*t*), *V*(*t*), *R*_*i*_(*t*), *J*_*ij*_(*t*), and *ϕ*_*ij*_(*t*).

#### The working equation

By definition, the internal molar concentration *c*_*i*_ [mol/cm^3^] is

(1) $$c_{i}\equiv \frac{N_{i}}{V}$$

where *N*_*i*_ is the number of moles of the molecule *i*. Thus, temporal variation of both the number of moles *N*_*i*_ and volume *V* may induce modifications of the internal concentration *c*_*i*_. The temporal variation of *c*_*i*_ is expressed by the partial time derivative ${\dot{c}}_{i}$ = *∂c*_*i*_/*∂t* of Eq. 1,

(2) $${\dot{c}}_{i}=\frac{1}{V}\left[{\dot{N}}_{i}-c_{i}\dot{V}\right].$$

The working Eq. 2 shows that the variation of the internal concentration is proportional to both the molar $\dot{N}$ [mol/s] and volume $\dot{V}$ [cm^3^/s] flow rates. Thus, to impart physical meaning to Eq. 2, we must introduce the physical phenomena inducing temporal variations of both *N*_*i*_ and *V*.

#### Molar variation ${\dot{N}}_{i}$

The number of moles *N*_*i*_ of molecule *i* in the vesicle lumen can change for two reasons: 1) molecular transport across the membrane and/or 2) chemical reaction kinetics generating or disassembling molecule *i*. In this respect, according to the reaction-diffusion equation, the molar flow rate in the vesicle is

(3) $${\dot{N}}_{i}=\int_{0}^{A}J_{ij}\left(\vec{r}\right)d\vec{r}+\int_{0}^{V}R_{i}\left(\vec{r}\right)d\vec{r}$$

where *J*_*ij*_$\left(\vec{r}\right)$ is the molar flux [mol/(s ⋅ cm^2^)] of molecule *i* across a small area *dA* of a specific membrane *j*, and *R*_*i*_$\left(\vec{r}\right)$ [mol/(s ⋅ cm^3^)] describes the reaction kinetics occurring in a small volume *dV* inside the vesicle. These two terms are integrated over the surface area *A* and the vesicle volume *V*, respectively. The reaction term *R*_*i*_ depends on the specific chemical reaction and can be either a source (*R* > 0) or a sink (*R* < 0) of molecules. For instance, *R* = −*ka* for a simple kinetics like $a\overset{k}{\to }b$.

To proceed further, we make two assumptions. First, the membrane composition is nanoscopically homogeneous. Therefore, the molar flux is uniform over the membrane surface; that is, *J*_*ij*_$\left(\vec{r}\right)$ = *J*_*ij*_. Second, the internal concentration is spatially uniform—that is, *c*_*i*_$\left(\vec{r}\right)$ = *c*_*i*_—which implies that the reaction term *R*_*i*_$\left(\vec{r}\right)$ = *R*_*i*_ is also uniform in the whole vesicle lumen. Thus, upon integration of the two terms, Eq. 3 becomes

(4) $${\dot{N}}_{i}=J_{ij}A+R_{i}V$$

where the first term is written as *ϕ*_*ij*_ = *J*_*ij*_*A* [mol/s].

#### Volume variation $\dot{V}$

To calculate the volume flow rate $\dot{V}$ [cm^3^/s], we note that a molecule crossing the membrane transports a small volume *dV* equal to the molar volume *M*_*i*_ [cm^3^/mol] divided by the Avogadro’s number *N*_*a*_ [mol^−1^], as well as a mass *MW*/*N*_*a*_ and a charge *ze*. Consequently, the molar flux *J*_*ij*_ of molecule *i* generates a volume flow rate *ϕ*_*V*,*ij*_ = *AM*_*i*_*J*_*ij*_ [cm^3^/(s ⋅ cm^2^)] across the vesicle surface, leading to swelling or shrinkage of the vesicle. Thus, the total volume flow rate *ϕ*_*V*,*j*_ generated by the fluxes of all permeable molecule is $\sum_{i}^{n}\phi_{V,ij}=A\sum_{i}^{n}M_{i}J_{ij}$. Assuming that the molar volume variation during a chemical reaction is very small with respect to the contribution of the volume flux across the membrane, the reaction term *R*_*i*_ is negligible. Thus, the total volume flow rate is

(5) $$\dot{V}=A\sum_{i}^{n}M_{i}J_{ij}.$$

Importantly, the molar flux *J*_*ij*_ couples the vesicle lumen with the external solution. In this respect, the vesicle is a nanoscopic chemical reactor fed by the molecular flux.

#### Dilute solution

To simplify the description presented above, we consider a dilute solution for which the molar fraction of water *x*_*w*_ = $N_{w}/\left(N_{w}+N_{s}\right)$ is very large with respect to the molar fraction of the *n* − 1 soluble species, that is, *x*_*w*_ ≫ *x*_*s*_. Indeed, under normal experimental conditions, the maximal solute molar fraction *x*_*s*_ is at least three orders of magnitude smaller than the water molar fraction *x*_*w*_. Thus, in a dilute solution, the solute contribution to the total volume is negligible—that is, *V* ≃ *V*_*w*_—and Eq. 5 simplifies to

(6) $$\dot{V}≃\overset{˙}{V_{w}}=AM_{w}J_{wj}$$

meaning that only the water flux across the membrane affects the vesicle volume.

#### Single vesicle dynamics

Now, by substitution of Eq. 4 in Eq. 2 and considering Eq. 6, we can write the *n* + 1 coupled differential equations describing the overall dynamics of the system:

(7) $$\begin{array}{l} {\dot{c}}_{i}=-\frac{c_{i}}{V}\dot{V}+\frac{A}{V}J_{ij}+R_{i}.\\ \dot{V}=AM_{w}J_{wj} \end{array}$$

By focusing on the three terms in the first equation, we observe that three physical phenomena can modify the internal concentrations *c*_*i*_. These phenomena are 1) the volume flow rate $\dot{V}$—that is, the vesicle swelling or shrinkage—induced by the water flux *J*_*wj*_; 2) the solute flux *J*_*ij*_ across the membrane; and 3) the chemical transformations *R*_*i*_ of the contained molecules. Importantly, all three terms are functions of the solute concentrations *c*_*i*_ (see next section) and, therefore, they are coupled.

### Passive transport

In the previous section, we disregarded any specific transport mechanism. Here, we introduce passive transport (either channel mediated or directly through the lipid bilayer), for which the molar flux *J*_*ij*_ is (5,6)

(8) $$J_{ij}=P_{ij}\left(c_{i}^{*}-c_{i}\right).$$

In Eq. 8, $c_{i}^{*}$ is the external concentration and *P*_*ij*_ is the permeability coefficient [cm/s] of molecule *i* through a membrane or protein channel *j*. Equation 8 shows that the concentration gradient *Δc*_*i*_ = $c_{i}^{*}$ − *c*_*i*_ drives the molar flux *J*_*ij*_ across the physical barrier, that is, the membrane. The sign of the gradient determines the direction of the molecular flux which is directed toward region with lower concentration. A positive (*Δc*_*i*_ > 0) and a negative (*Δc*_*i*_ < 0) gradient determine influx and outflux of molecules, respectively. The equilibrium is reached when the solute gradient dissipates, that is, *Δc*_*i*_ = 0.

Next, we consider that in a dilute solution, the molar flux of water *J*_*wj*_ depends on the total solute concentration gradient *Δc*_*s*_ = $c_{s}^{*}$ − *c*_*s*_ (7) as follows:

(9) $$J_{wj}≃P_{wj}\left[\frac{\Delta P_{M}}{RT}-\left(c_{s}^{*}-c_{s}\right)\right]$$

where the constants $c_{s}=\sum_{i}^{n-1}c_{i}$ and $c_{s}^{*}=\sum_{i}^{m-1}c_{i}^{*}$ are the total internal and external solute concentrations, respectively, and *ΔP*_*M*_ is the hydrostatic (or mechanical) pressure opposing the osmotic pressure, that is, *Π* ≃ *RTΔc*_*s*_. Importantly, in our description, we consider only osmotic upshifts, that is, positive gradients *Δc*_*s*_ > 0 leading to vesicle shrinkage. From Eq. 9, we observe that 1) the solute concentration gradient *Δc*_*s*_ induces a water flux across the membrane, 2) the water flux is directed toward the region with higher solute concentration, and 3) the mechanical resistance of the membrane (*ΔP*_*M*_ > 0) slows down the water flux. Because a typical lipid membrane can mechanically sustain very small concentration gradients up to 0.1 *μ*M (8,9), for values above 0.1 *μ*M, the membrane freely deforms without opposing mechanical resistance to the osmotic pressure; that is, *ΔP*_*M*_ ≪ *Π*. Thus, we set *ΔP*_*M*_ ≃ 0 and rewrite Eq. 7 in the dilute solution limit

(10) $$\begin{array}{l} {\dot{c}}_{i}=\frac{A}{V}\left[c_{i}M_{w}P_{wj}\left(c_{s}^{*}-\sum_{i}^{n-1}c_{i}\right)+P_{ij}\left(c_{i}^{*}-c_{i}\right)\right]+R_{i}\\ \dot{V}≃-AM_{w}P_{wj}\left(c_{s}^{*}-\sum_{i}^{n-1}c_{i}\right) \end{array}$$

where *i* = *n* − 1 identifies all molecular species enclosed by the vesicle except for water. Indeed, we can show that, for a dilute solution, the water concentration is time independent (${\dot{c}}_{w}$ ≃ 0). Importantly, both $c_{i}^{*}$ and $c_{s}^{*}$ are constant. Equation 10 demonstrates that the relaxation dynamics of the volume *V* and internal concentrations *c*_*i*_ are driven by the concentration gradients (*Δc*_*i*_ and *Δc*_*s*_) across the membrane. Furthermore, the relaxation dynamics depends 1) on the magnitude of the concentration gradients (||*Δc*_*i*_|| and ||*Δc*_*s*_||); 2) on the physiochemical properties of the membrane, such as the surface area *A* and the permeability coefficient *P*_*ij*_; and 3) on the chemical properties of the contained molecules, such as *P*_*ij*_ and *R*_*i*_. Equation 10 is the most important outcome of this work. Indeed, the equations generalize the dynamic description of any experimental system that is well approximated by a closed compartment delimited by a permeable membrane to account for the effects of volume variation. Also, the model can comprise both multiple permeable and impermeable molecules as well as any chemical reaction between the solute molecules.

#### Problem rescaling

To better grasp the physics of the system and to prepare the equations for numerical solution, we aim to obtain dimensionless equations (10). To this end, we transform the variables as follows:

(11) $${\bar{c}}_{i}=\frac{c_{i}}{c_{s}^{*}},\;\;\;\bar{V}=\frac{V}{V_{0}},\;\;\;\bar{t}=\frac{t}{t_{c}}$$

where *t*_*c*_ [s] is an arbitrary characteristic time and *V*_0_ [cm^3^] is the vesicle volume at time *t* = 0. Thus, the transformed concentrations and volume ∈ [0, 1] and $\bar{V}$(0) = 1. By substituting these new variables in Eqs. 10 and by setting *t*_*c*_ = ${\left(\frac{A}{V_{0}}P_{wj}M_{w}c_{s}^{*}\right)}^{-1}$ (see Appendix B), we obtain the desired dimensionless equations

(12) $$\begin{array}{l} {\dot{\bar{c}}}_{i}=\frac{1}{\bar{V}}\left[-{\bar{c}}_{i}\left(\overset{n-1}{\sum_{i}}{\bar{c}}_{i}-1\right)+\lambda_{ij}\left(1-\frac{{\bar{c}}_{i}}{\gamma_{i}}\right)\right]+{\bar{R}}_{ij}\\ \dot{\bar{V}}≃\overset{n-1}{\sum_{i}}{\bar{c}}_{i}-1\\ \lambda_{ij}=\frac{P_{ij}}{P_{wj}}\frac{\gamma_{i}}{M_{w}c_{s}^{*}},\;\gamma_{i}=\frac{c_{i}^{*}}{c_{s}^{*}} \end{array}$$

where ${\bar{R}}_{ij}$ is a dimensionless reaction term. We note that the ratio $\left({\bar{c}}_{i}/\gamma_{i}\right)$ is equal to the ratio $\left(c_{i}/c_{i}^{*}\right)$ between the internal and external concentration of molecule *i*. Remarkably, the dynamics of the system (or dynamic state of the system) is completely defined by a set of 2(*n* − 1) dimensionless parameters {*λ*_*ij*_, *γ*_*i*_} and *n* starting conditions $\left\{{\bar{c}}_{i}\left(0\right),\bar{V}\left(0\right)\right\}$. We can simulate the whole set of possible dynamic behaviors of the system (shape of the relaxation curves) by modifying these parameters with given starting conditions. If all *λ*_*ij*_ are equal to zero, only one dynamic regime is observed, whereas the dynamic complexity of the system increases with the number of parameters.

### Yeast cell relaxation dynamics

#### Problem redefinition

To describe the volume and pH dynamics of a yeast cell upon osmotic upshift, we followed the procedure applied to vesicles but with additional assumptions. We describe the yeast cell as a spherical shell (11) that contains solute molecules, organelles, and macromolecules. The latter occupies the so-called nonosmotic volume *b* (11, 12, 13), inaccessible to solutes. The model cell is delimited by a spherical model membrane with variable volume *V* and surface area $A=V^{\frac{2}{3}}{\left(\frac{3}{4\pi }\right)}^{\frac{2}{3}}$, reproducing the most relevant features of both the plasma membrane and the cell wall, i.e., semipermeability and elasticity.

#### Dynamic description

The aforementioned hypotheses are incorporated in our description as follows. First, we redefine the internal solute concentration accounting for the nonosmotic volume *b*,

(13) $$c_{i}\equiv \frac{N_{i}}{V-b}.$$

Second, we introduce a term for elasticity that generates a hydrostatic (or turgor) pressure *ΔP*_*M*_ (11,14, 15, 16),

(14) $$\Delta P_{M}=\epsilon \frac{\Delta V}{V_{r}}.$$

Here, *ε* [MPa] is the volumetric elastic modulus and *ΔV* = *V*_*r*_ − *V* is the relative volume variation with respect to the reference volume *V*_*r*_, which we set to the cell volume at zero turgor, that is, *V*_*r*_ = $V_{\left(\Delta P_{M}=0\right)}$. Thus, by exploiting the redefined concentration *c*_*i*_ and the turgor pressure *ΔP*_*M*_, we derive the following system of differential equations describing the model cell relaxation dynamics upon osmotic shock:

(15) $$\begin{array}{l} {\dot{c}}_{i}=-\frac{A}{V-b}\left[c_{i}\dot{V}-P_{ij}\left(c_{i}^{*}-c_{i}\right)\right]+R_{i}\\ \dot{V}≃AM_{w}P_{wj}\left[\frac{\epsilon }{RT}\frac{\text{Δ}V}{V_{r}}-\left(c_{s}^{*}-\sum_{i}^{n-1}c_{i}\right)\right]\\ A={\left(\frac{3}{4\pi }\right)}^{\frac{2}{3}}V^{\frac{2}{3}}. \end{array}$$

The balance between the two terms inside the square brackets (see second equation of Eq. 15) determines whether the volume shrinks or swells upon relaxation. The first term depends on the elasticity of the model membrane, whereas the second term is the solute concentration gradient *Δc*_*s*_ across the membrane, as defined in the previous section. We observe that in our experiments (presented in this issue (4)), the cellular volume is always larger than the reference volume *V*_*r*_, that is, *V* > *V*_*r*_. Thus, *ΔV* = *V*_*r*_ − *V* is negative, meaning that the model membrane opposes (slows down) swelling and favors (speeds up) shrinking of the cell similarly to a bicycle inner tube. Following the same analogy, pumping air inside the tube would correspond to an osmotic downshift (*Δc*_*s*_ > 0) and sucking air out to an osmotic upshift (*Δc*_*s*_ < 0). We also note that for yeast, in contrast to the vesicle description, the surface area *A* is variable.

#### Problem scaling

Analogously to vesicles, we derive dimensionless equations for the yeast model cell but with the difference that the volume *V* is scaled with respect to the zero-turgor volume *V*_*r*_ instead of the volume at time 0 *V*_0_:

(16) $$\bar{V}=\frac{V}{V_{r}}.$$

Thus, the characteristic time becomes *t*_*c*_ = ${\left(\frac{A_{r}}{V_{r}}P_{wj}M_{w}c_{s}^{*}\right)}^{-1}$, and the system of dimensionless differential equations is

(17) $$\begin{array}{l} {\dot{\bar{c}}}_{i}=-\frac{1}{\bar{V}-\bar{b}}\left[{\bar{c}}_{i}\dot{\bar{V}}-\lambda_{ij}\left(1-\frac{{\bar{c}}_{i}}{\gamma_{i}}\right)\right]+{\bar{R}}_{ij}\\ \dot{\bar{V}}≃{\bar{V}}^{\frac{2}{3}}\left[\bar{\Theta }\left(1-\bar{V}\right)+\sum_{i}^{n-1}{\bar{c}}_{i}-1\right]\\ \lambda_{ij}=\frac{P_{ij}}{P_{wj}}\frac{\gamma_{i}}{M_{w}c_{s}^{*}},\;\gamma_{i}=\frac{c_{i}^{*}}{c_{s}^{*}},\;\bar{b}=\frac{b}{V_{r}},\;\bar{\Theta }=\frac{\epsilon }{RTc_{s}^{*}}. \end{array}$$

We note that the dynamics of the model cell with respect to that of vesicles depends on two additional parameters, which are $\bar{b}$ and $\bar{\Theta }$. These parameters, which originate from the additional assumptions made for yeast (nonosmotic volume and semipermeability and elasticity of the cell envelope), add complexity to the relaxation dynamics of the model cell.

## Results

### Osmotic-shock perturbation

From now on, the mathematical tools that we built are used to describe the relaxation dynamics of a vesicle or cell perturbed by an osmotic upshift, that is, an increase of the external solute concentration $c_{s}^{*}$. First, we assume that for times *t* < 0, the vesicle or cell is in a stationary state. The vesicle is in a stationary state if *Δc*_*s*_ = 0 and *Δc*_*i*_ = 0 $\forall_{i}$. The cell is in a stationary state if the turgor pressure equals the osmotic pressure, that is, $\left(\epsilon /RT\right)\left(\Delta V/V_{r}\right)$ = *Δc*_*s*_ and *Δc*_*i*_ = 0 $\forall_{i}$. In the stationary time regime, the dynamics is governed by statistical fluctuations *δΔc*_*i*_ of the molar concentration gradients around the average values *Δc*_*i*_. Second, at time *t* = 0, we apply an osmotic upshift. Thus, for times *t* > 0, the concentration gradient governs the dynamics of the vesicle or cell, which relaxes to equilibrium under the constraints given in Eq. 10 (or Eq. 15). The relaxation dynamics is described by the solutions *c*_*i*_ and *V* of Eq. 10 (or Eq. 15), which are calculated with the well-defined starting conditions {*c*_*i*,0_, $c_{i,0}^{*}$, *V*_0_} and parameters {*A*, *P*_*ij*_, *P*_*wj*_, *M*_*w*_, *R*_*i*_, (*ε*, *b*, *V*_*r*_)}. Because the relaxation dynamics is univocally determined by the chemical composition of the internal and external solutions and by the vesicle or cell physiochemical properties (see above), the relaxation kinetic curves are fingerprints of the system’s physiochemical properties.

### Calculation of pH and calcein fluorescence emission

To compare the osmotic-upshift kinetic experiments with the model predictions, we calculate the two physical quantities obtained from our kinetic experiments. These are 1) the pH of the vesicle or cell lumen and 2) the normalized fluorescence emission intensity *F*(*t*)/*F*(0) of calcein encapsulated in the vesicle at self-quenching concentration (17). The first quantity, pH, is sensitive to the permeation of a weak acid or base across the membrane, whereas the second quantity, *F*(*t*)/*F*(0), responds to the volume variation. The internal pH is easily calculated from the proton concentration, that is, pH = −log_10_[H^+^]. The normalized fluorescence intensity of calcein *F*(*t*)/*F*(0) is obtained by modifying the Stern-Volmer (equation 18) according to

(18) $$\frac{F\left(t\right)}{F\left(0\right)}=\frac{1+K_{SV}c\left(0\right)}{1+K_{SV}c\left(t\right)}$$

where *K*_*SV*_ ≃ 100 M^−1^ is the dynamic self-quenching constant of calcein (17) and *c*(0) and *c*(*t*) are calcein concentrations before and after the osmotic shock, respectively. We note that both quantities are calculated differently if an ensemble of vesicles with heterogeneous size distribution is considered (see Appendix B).

### Model examples: Vesicles

Here, we present examples on the construction of the dynamic model for description of real experiments; that is, we show how to build up the equations describing the vesicle dynamics. We focus on the osmotic-upshift kinetic experiments performed on lipid vesicles (presented in this issue (4)). We stress that for the numerical solution, the equations reported in the following sections must be rescaled as described above and in Appendix A. Our aim is to provide everyone with essential instructions to set up the modeling tools and to recognize the fingerprint of the vesicle physiochemical properties from the relaxation curves.

#### Problem definition

We assume that before the osmotic upshock, at time *t* < 0, the vesicle lumen is filled with a water solution at pH 7 containing 90 mM potassium phosphate (KPi) and 10 mM of the fluorophore calcein (17). The external water solution, also at pH 7, contains 100 mM KPi. At time *t* = 0, we osmotically upshift the vesicle solution by mixing it with a 100 mM KPi water solution (pH 7) containing one or more additional osmolytes such as KCl, glycerol, a weak acid or base, a mixture of weak acid and base, etc.

#### Impermeable solute

We start by constructing the model for vesicles osmotically perturbed with an impermeable osmolyte having concentration $c_{6}^{*}$. The concentration $c_{6}^{*}$ is the total osmolyte concentration upon dissolution in the water solution. In this respect, upon osmotic upshift with a salt such as KCl, the total osmolyte concentration is $c_{6}^{*}$ = 2[KCl] = [K^+^] + [Cl^−^]. First, we identify the most abundant molecular species in the vesicle lumen, which at pH 7 are [H_2_O] = *c*_1_, [calcein] = *c*_2_, [H_2_PO_4_^−^] = *c*_3_, [HPO_4_^2−^] = *c*_4_, and [H^+^] = *c*_5_. Second, we identify the prevalent chemical equilibria between the molecular species in solution, which for the KPi buffer at pH 7 are

(19) $$\begin{array}{l} {\text{H}}_{2}{\text{PO}}_{4}^{-}\overset{k_{1}}{\underset{k_{-1}}{⇌}}{\text{HPO}}_{4}^{2-}+{\text{H}}^{+}\\ c_{3}\overset{k_{1}}{\underset{k_{-1}}{⇌}}c_{4}+c_{5}. \end{array}$$

Third, we set the starting internal concentrations, which are either known (*c*_2,0_ = 10 mM and *c*_6,0_ = 0) or calculated by using the Henderson-Hasselbach equation and the definition of pH: {*c*_2,0_, *c*_3,0_, *c*_4,0_, *c*_5,0_}. Also, we calculate the external solute concentration $c_{s}^{*}=c_{3}^{*}+c_{4}^{*}+c_{5}^{*}+c_{6}^{*}$, which is constant in time, by knowing the concentration of the impermeable osmolyte $c_{6}^{*}$, KPi (100 mM), and the external pH. Fourth, we assume that only water is permeable on the observed timescales; that is, *P*_1_ > 0 and {*P*_2_, *P*_3_, *P*_4_, *P*_5_, *P*_6_} = 0 for *t* ∈ [0.001–10] s. Thus, according to Eq. 10, we write the 5 + 1 differential equations describing the dynamics of the system as

(20) $$\begin{array}{l} {\dot{c}}_{1}=0\\ {\dot{c}}_{2}=-\frac{1}{V}c_{2}\dot{V}\\ {\dot{c}}_{3}=-\frac{1}{V}c_{3}\dot{V}-k_{1}c_{3}+k_{-1}c_{4}c_{5}\\ {\dot{c}}_{4}=-\frac{1}{V}c_{4}\dot{V}+k_{1}c_{3}-k_{-1}c_{4}c_{5}\\ {\dot{c}}_{5}=-\frac{1}{V}c_{5}\dot{V}+k_{1}c_{3}-k_{-1}c_{4}c_{5}\\ \dot{V}=-AM_{1}P_{1}\left(c_{s}^{*}-c_{2}-c_{3}-c_{4}-c_{5}\right). \end{array}$$

We observe that 1) all equations from the second to the fifth contain a volume term $-\frac{1}{V}c_{i}\dot{V}$; 2) the third through fifth equations, which describe the dissociation of KPi, also contain the chemical kinetic terms coupling *c*_3_, *c*_4_, and *c*_5_; 3) the second equation only contains the volume term because calcein does not participate in any chemical reaction; 4) all equations are coupled by the concentration gradient *Δc*_*s*_ throughout the $\dot{V}$ term; and 5) no transport term is found because all molecules except for water are impermeable.

Now, to decrease the number of free parameters, we introduce the acid-base dissociation constant *K*_1_ = $\left(k_{1}/k_{-1}\right)=\left(c_{4}c_{5}/c_{3}\right)$ and rewrite the previous equations as follows:

(21) $$\begin{array}{l} {\dot{c}}_{1}=0\\ {\dot{c}}_{2}=-\frac{1}{V}c_{2}\dot{V}\\ {\dot{c}}_{3}=-\frac{1}{V}c_{3}\dot{V}-k_{1}\left(c_{3}-\frac{c_{4}c_{5}}{K_{1}}\right)\\ {\dot{c}}_{4}=-\frac{1}{V}c_{4}\dot{V}+k_{1}\left(c_{3}-\frac{c_{4}c_{5}}{K_{1}}\right)\\ {\dot{c}}_{5}=-\frac{1}{V}c_{5}\dot{V}+k_{1}\left(c_{3}-\frac{c_{4}c_{5}}{K_{1}}\right)\\ \dot{V}=-AM_{1}P_{1}\left(c_{s}^{*}-c_{2}-c_{3}-c_{4}-c_{5}\right). \end{array}$$

The dissociation constant *K*_1_ [M^−1^] is calculated from the *pK*_*a*_ of KPi, which is *pK*_1_ = 7.21. The surface area *A* and the starting volume *V*_0_ of the sphere are calculated from the radius *r*_0_. The water molar volume is known: *M*_1_ = 18 cm^3^/mol. The microscopic rate constants *k*_1_ and *k*_−1_ are usually on the order of (10^5^–10^6^) s^−1^ (19). The permeability coefficient of water *P*_1_ is ∼10^−3^ cm/s for a typical lipid membrane composition (1). Thus, the numerical solutions *c*_*i*_ and *V* of the rescaled equations are computed by using the MATLAB (The MathWorks, Natick, MA) *ode15s* solver upon linearization of the equations with the Jacobian matrix.

**Figure 1:**
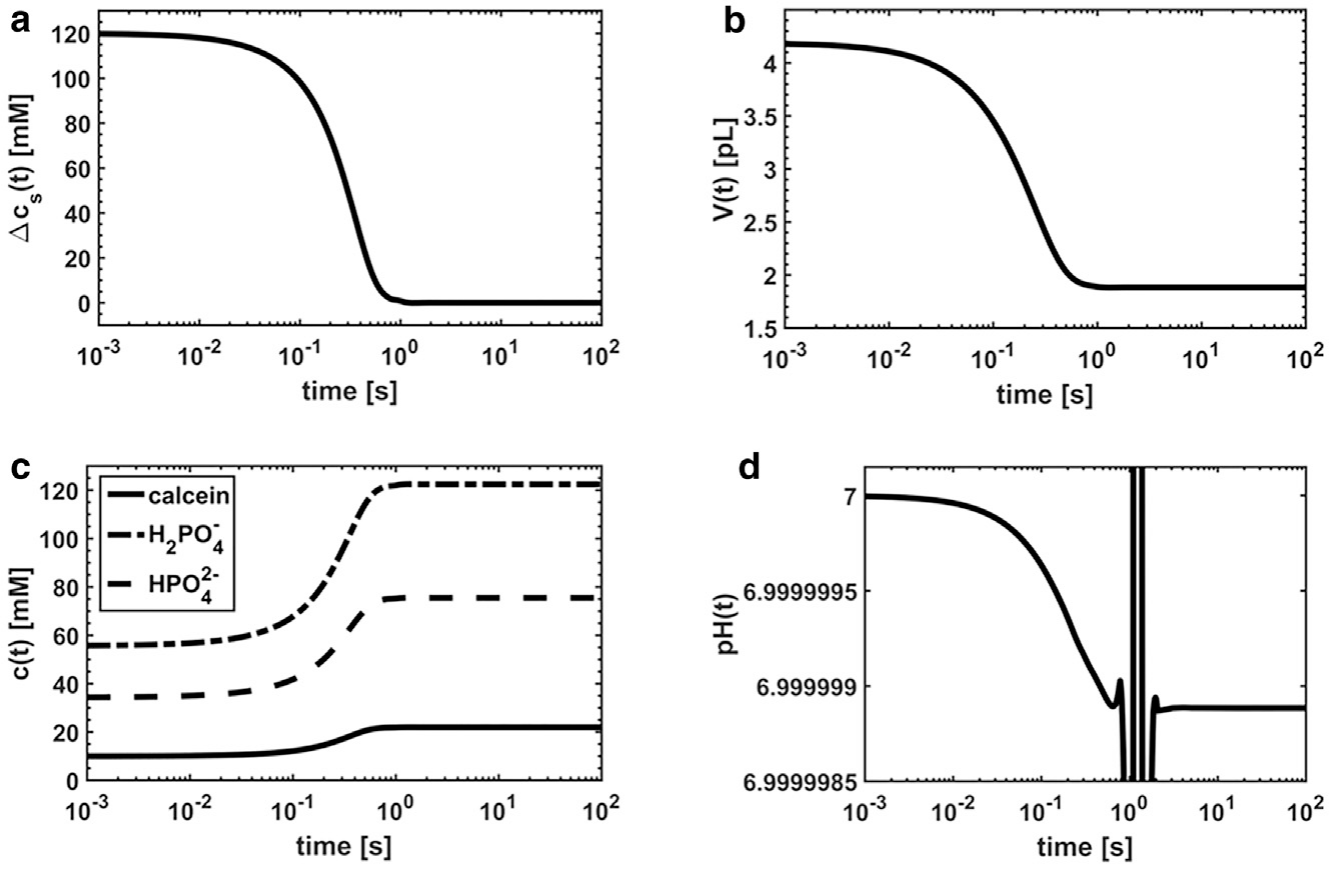
Simulated curves for an impermeable solute. (*a*) Variation of the solute concentration gradient across the membrane *Δc*_*s*_(*t*) is shown. (*b*) Vesicle volume variation *V*(*t*) is shown. (*c*) Variation of the internal solute concentrations *c*_*i*_(*t*) is shown. (*d*) Variation of the internal pH is shown. The following parameters were used for calculations: *pK*_1_ = 7.21, *M*_1_ = 18 cm^3^/mol, pH_0_ = ${\text{pH}}_{0}^{*}$ = 7.0, [KPi] = 90 mM, [KPi]^∗^ = 100 mM, [calcein] = 10 mM, *k*_1_ = 10^6^ s^−1^, *K*_*SV*_ = 10^2^ M^−1^, $c_{6}^{*}$ = 120 mM, *r*_0_ = 100 nm, and *P*_1_ = 0.003 cm/s. For calculation of pH(*t*), we set [KPi] = 100 mM and [calcein] = 0 M.

Next, we discuss the most important features of the vesicle dynamics as a function of the physical parameters of the system as shown in Fig. 1. First, the relaxation dynamics lasts until the driving force is completely dissipated at ∼10^0^ s (that is, the solute concentration gradient is 0 (Fig. 1 *a*)). Second, the vesicle volume shrinks to approximately 50% (Fig. 1 *b*), thereby inducing an equivalent relative increase of the internal solute concentrations (Fig. 1 *c*). Third, the ratio between the concentration of H_2_PO_4_^−^ and HPO_4_^2−^ is constant and defined by the weak acid dissociation constant *K*_1_. Fourth, the pH variation induced by the volume reduction is below 0.0001%, owing to the buffering capacity of KPi (Fig. 1 *d*). The fluctuations at around 1 s observed in the pH relaxation curve are instabilities of the numerical solution related to the numerical precision of the software.

**Figure 2:**
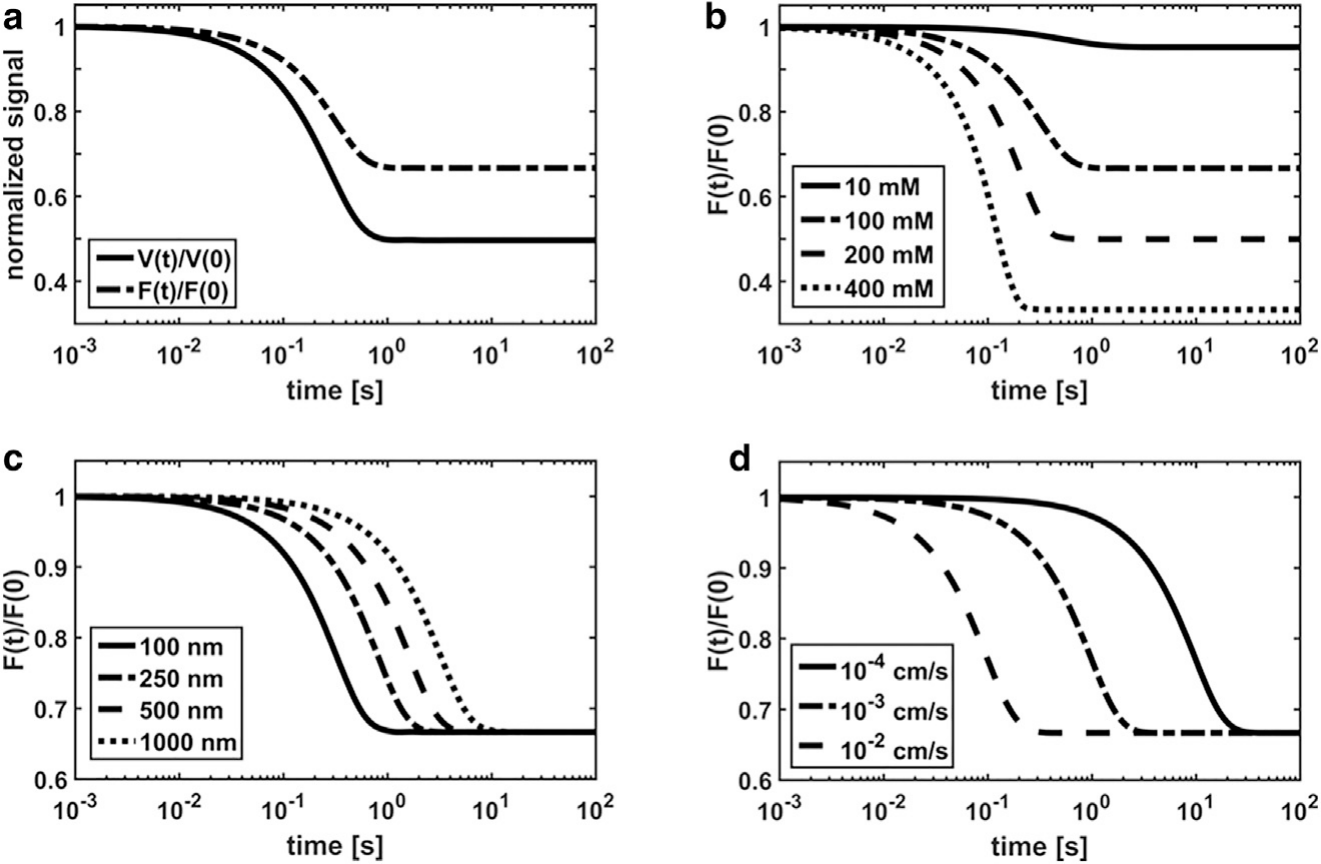
Simulated curves for an impermeable solute. (*a*) Comparison of normalized volume *V*(*t*)/*V*(0) and calcein fluorescence intensity *F*(*t*)/*F*(0) is shown. Variation of *F*(*t*)/*F*(0) is shown as a function of (*b*) the solute concentration gradient *Δc*_*s*_, (*c*) the vesicle radius *r*_0_, and (*d*) the water permeability coefficient *P*_*wj*_. The following parameters were used for calculations: *pK*_1_ = 7.21, *M*_1_ = 18 cm^3^/mol, pH_0_ = ${\text{pH}}_{0}^{*}$ = 7.0, [KPi] = 90 mM, [KPi]^∗^ = 100 mM, [calcein] = 10 mM, *k*_1_ = 10^6^ s^−1^, *K*_*SV*_ = 10^2^ M^−1^, $c_{6}^{*}$ = 100 mM, *r*_0_ = 100 nm, and *P*_1_ = 0.003 cm/s. The last three parameters ($c_{6}^{*}$, *r*_0_, *P*_1_) were modified according to the figure legends.

To get insight into the effects of the vesicle properties on the relaxation kinetics, we focus on the measured ratio *F*(*t*)/*F*(0). In previous works (1,2), the experimental relaxation data *F*(*t*)/*F*(0)_*exp*_ were fitted with the volume ratio *V*(*t*)/*V*(0). In Fig. 2 *a*, we compare the calculated vesicle volume *V*(*t*)/*V*(0) with the calcein fluorescence intensity *F*(*t*)/*F*(0), both normalized to 1 at time 0. We observe the difference between the two simulated relaxation curves. Therefore, we strongly recommend using the calculated normalized fluorescence intensity *F*(*t*)/*F*(0) instead of the normalized volume *V*(*t*)/*V*(0) for fitting of the experimental data. In the remaining three panels of Fig. 2, we show how variations of the magnitude of the driving force *Δc*_*s*_, the vesicle radius *r*_0_, and the permeability coefficients *P*_*ij*_ affect the measured relaxation rates. The increase of the solute concentration gradient *Δc*_*s*_ increases the relaxation rate (note that the vesicles reach equilibrium earlier for larger gradients; Fig. 2 *b*) and the maximal vesicle shrinkage. Importantly, variations of the permeability coefficient and vesicle radius have an equivalent but opposite effect on the relaxation rate (compare Fig. 2 *c* and Fig. 2 *d*); that is, the increase of the radius and decrease of the permeability coefficient induce a decrease of the relaxation rates and vice versa. Therefore, it is crucial to accurately determine the vesicle radius for a correct estimation of permeability coefficients. Interestingly, we observe that variation of the physical parameters in Fig. 2 has no effect on the shape of the curves (that is, on the dynamics type or character of the relaxation kinetics), but only on the relaxation rate (that is, on “the speed to re-establish the equilibrium”). In this respect, more complex dynamics are observed upon the introduction of permeable solutes (see below).

#### Permeable weak acid

In the second example, we osmotically perturb the vesicle solution with a weak acid AH that at pH 7 dissociates to the following chemical equilibrium

(22) $$\begin{array}{l} \text{AH}\overset{k_{2}}{\underset{k_{-2}}{⇌}}{\text{A}}^{-}+{\text{H}}^{+}\\ c_{6}\overset{k_{2}}{\underset{k_{-2}}{⇌}}c_{7}+c_{5}. \end{array}$$

**Figure 3:**
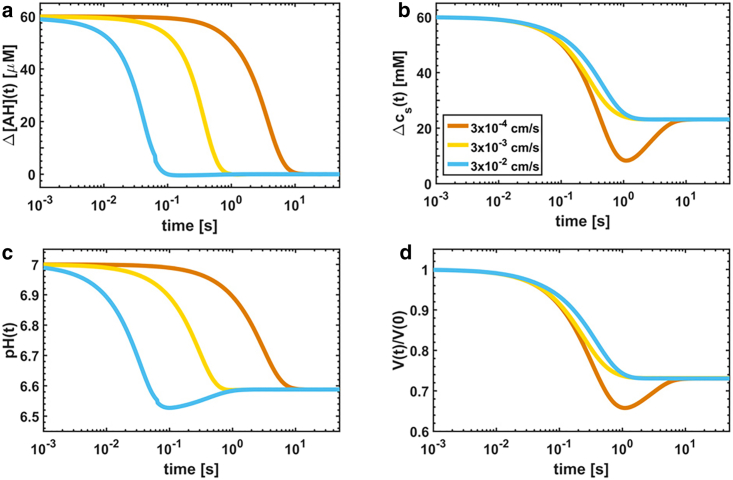
Simulated curves for permeable weak acids. The permeability coefficient of water is fixed to *P*_1_ = 0.003 cm/s, whereas the weak acid permeability *P*_6_ was modified as indicated in the figure legend. The time evolution of (*a*) the acid concentration gradient *Δ*[AH] of AH and (*b*) the solute concentration gradient *Δc*_*s*_ is shown. The relaxation dynamics of (*c*) internal pH and (*d*) normalized volume *V*(*t*)/*V*(0) are shown. The following parameters were used for calculations: *pK*_1_ = 7.21, *pK*_2_ = 4, *M*_1_ = 18 cm^3^/mol, pH_0_ = ${\text{pH}}_{0}^{*}$ = 7.0, [KPi] = 90 mM, [KPi]^∗^ = 100 mM, [calcein] = 10 mM, *k*_1_ = 10^6^ s^−1^, *K*_*SV*_ = 10^2^ M^−1^, $c_{6}^{*}+c_{7}^{*}$ = 60 mM, *r*_0_ = 100 nm, and *P*_1_ = 0.003 cm/s. For calculation of pH(*t*), we set [KPi] = 100 mM and [calcein] = 0 M. To see this figure in color, go online.

For simplicity, we do not consider here the counterion (Na^+^, K^+^, or Li^+^) that is released in the solution upon addition of the weak acid salt. However, the presence of the ion, which contributes to the total solute gradient *Δc*_*s*_, is taken into account in the analysis of the measured relaxation kinetics (see the accompanying study (4)). We assume that besides water, only the neutral species AH is permeable through the membrane, that is, *P*_6_ > 0. Thus, the most abundant chemical species in the vesicle lumen before the osmotic shock are [H_2_O] = *c*_1_, [calcein] = *c*_2_, [H_2_PO_4_^−^] = *c*_3_, [HPO_4_^2−^] = *c*_4_, and [H^+^] = *c*_5_. However, after the osmotic shock, two additional chemical species [AH] = *c*_6_ and [A^−^] = *c*_7_ are present in the lumen because AH permeates the membrane and dissociates according to Eq. 22. Therefore, the starting internal concentrations {*c*_2,0_, *c*_3,0_, *c*_4,0_, *c*_5,0_} are calculated as described in the previous example. Also, we know that *c*_6,0_ = *c*_7,0_ = 0. The external solute concentration is $c_{s}^{*}=c_{3}^{*}+c_{4}^{*}+c_{5}^{*}+c_{6}^{*}+c_{7}^{*}$, and the permeability coefficients are {*P*_1_, *P*_6_} > 0 and {*P*_2_, *P*_3_, *P*_4_, *P*_5_, *P*_7_} = 0. Thus, the system of 7 + 1 equations describing the vesicle dynamics is

(23) $$\begin{array}{l} {\dot{c}}_{1}=0\\ {\dot{c}}_{2}=-\frac{1}{V}c_{2}\dot{V}\\ {\dot{c}}_{3}=-\frac{1}{V}c_{3}\dot{V}-k_{1}\left(c_{3}-\frac{c_{4}c_{5}}{K_{1}}\right)\\ \dot{c}{}_{4}=-\frac{1}{V}c_{4}\dot{V}+k_{1}\left(c_{3}-\frac{c_{4}c_{5}}{K_{1}}\right)\\ {\dot{c}}_{5}=-\frac{1}{V}c_{5}\dot{V}+k_{1}\left(c_{3}-\frac{c_{4}c_{5}}{K_{1}}\right)+k_{2}\left(c_{6}-\frac{c_{7}c_{5}}{K_{2}}\right)\\ {\dot{c}}_{6}=-\frac{1}{V}c_{6}\dot{V}-k_{2}\left(c_{6}-\frac{c_{7}c_{5}}{K_{2}}\right)+\frac{A}{V}P_{6}\left(c_{6}^{*}-c_{6}\right)\\ {\dot{c}}_{7}=-\frac{1}{V}c_{7}\dot{V}+k_{2}\left(c_{6}-\frac{c_{7}c_{5}}{K_{2}}\right)\\ \dot{V}=-AM_{1}P_{1}\left(c_{s}^{*}-c_{2}-c_{3}-c_{4}-c_{5}-c_{6}-c_{7}\right). \end{array}$$

where *K*_2_ is the dissociation constant obtained from the weak acid *pK*_*a*_. With respect to the previous example (Eq. 21), we observe 1) the presence of the transport term in the sixth equation, which chemically couples the vesicle lumen with the external solution; and 2) the chemical coupling of the third through seventh equations by means of the proton concentration *c*_5_, which describes the buffering capacity of KPi that counteracts the acidification of the lumen induced by the weak acid influx. Indeed, the weak acid, which carries a proton across the membrane and then releases it in the vesicle lumen, effectively acts as a proton source, whereas KPi acts as a sink of protons. The detailed balance between the source and the sink terms determines the overall pH variation. The opposite behavior is observed for a weak base BH^+^, for which the equations must be modified accordingly. In Fig. 3, the relaxation dynamics are calculated by varying the permeability coefficient of the weak acid with respect to that of water, which we keep constant. In the upper panels, we display the relaxation kinetics of the acid (Fig. 3 *a*) and of the solute concentration gradients (Fig. 3 *b*) that drive the relaxation of the internal pH (Fig. 3 *c*) and volume (Fig. 3 *d*), respectively. We observe that the acid flux, that is, the transport term in the sixth equation, introduces extra features in the relaxation curves with respect to the previous example (compare Fig. 3 *d* with Fig. 2 *a*). Namely, three different dynamic regimes or behaviors are observed with respect to the single dynamic regime displayed upon perturbation with an impermeable osmolyte.

Dynamic regime #1: When the acid permeability is at least one order of magnitude larger than the water permeability (that is, *P*_6_ ≥ 10 × *P*_1_) (*blue curves*), the acid gradient *Δ*[AH] dissipates faster than the solute gradient *Δc*_*s*_ (in Fig. 3 *a*, the acid gradient *Δ*[AH] stops decreasing at ∼10^−1^ s, whereas in Fig. 3 *b*, the solute gradient *Δc*_*s*_ reaches a minimum at ∼10^0^ s). Also, we observe that the relaxation of pH and volume occurs simultaneously with the concentration gradients (compare Fig. 3 *a* with Fig. 3 *c* and Fig. 3 *b* with Fig. 3 *d*), showing that the gradients dissipate as a result of the acid and water flux, respectively. Remarkably, the blue curve in Fig. 3 *c* shows an increase of pH in the time interval from 10^−1^ s and 10^0^ s. Indeed, the volume shrinkage, which follows in time the dissipation of the gradient *Δ*[AH], increases the internal acid concentration above the external value ([AH] > [AH]^∗^). Consequently, to re-establish the equilibrium (that is, *Δ*[AH] = 0), the acid flows out of the lumen, causing the observed increase of pH. Furthermore, from Fig. 3 *b*, it may appear that the solute gradient does not dissipate completely but stabilizes to *Δc*_*s*_ ≃ 22 mM. This is explained by the large amount of acid (∼22 mM) diffusing into the vesicle lumen to equilibrate the concentration gradient *Δ*[AH] of ∼60 *μ*M and compensate the buffering capacity of KPi. Therefore, the internal solute concentration *c*_*s*_ increases from the starting value of 100 mM to ∼122 mM, thus lowering the gradient *Δc*_*s*_ from 60 mM to (160 − 122 mM) 38 mM.
Dynamic regime #2: When the acid and water permeability coefficients are similar (that is, *P*_6_ ≃ *P*_1_) (see the *yellow curve*), the acid and solute gradients relax on the same timescale, both ending at ∼10^0^ s (compare *upper panels*). Thus, the pH (acid inflow) and volume (water outflow) also have similar relaxation times (compare *bottom panels*).
Dynamic regime #3: When the acid permeability is slower than that of water (that is, *P*_6_ ≤ 0.1 × *P*_1_) (*orange curves*), the solute gradient dissipates faster than the acid gradient (compare the *upper panels* in which *Δc*_*s*_ stops decreasing at ∼10^0^, whereas *Δ*[AH] stops at ∼10^1^). Thus, the volume decreases faster than the pH (compare the *bottom panels*). Interestingly, for times longer than 10^0^ s, the volume swells (Fig. 3 *d*) to compensate for the increase of the internal solute concentration (Fig. 3 *b*) induced by the acid influx.

We observe that knowledge of both pH(*t*) and *V*(*t*) is required to differentiate between the three dynamic regimes. Indeed, from pH-only data, regime 2 is almost indistinguishable from regime 3 (compare the shape of the *yellow* and *orange curves* in Fig. 3 *c*). Furthermore, regime 1 and 2 are hardly separated from volume-only data (compare the shape of the *yellow* and *blue curves* in Fig. 3 *d*). On the contrary, by looking at both pH and volume data, we can immediately assess the dynamic regime of the system.

More generally, the transition between the three dynamic regimes observed for weak acids depends on one dimensionless parameter, that is, *λ*_6_, appearing in the rescaled equations (see Appendix A). By looking at the definition of *λ* (see Eq. 12), we write the dependence of *λ*_6_ from the physical parameters of the system

(24) $$\lambda_{6}\propto \frac{P_{6}}{P_{1}}\frac{c_{6}^{*}}{c_{s}^{*2}}.$$

**Figure 4:**
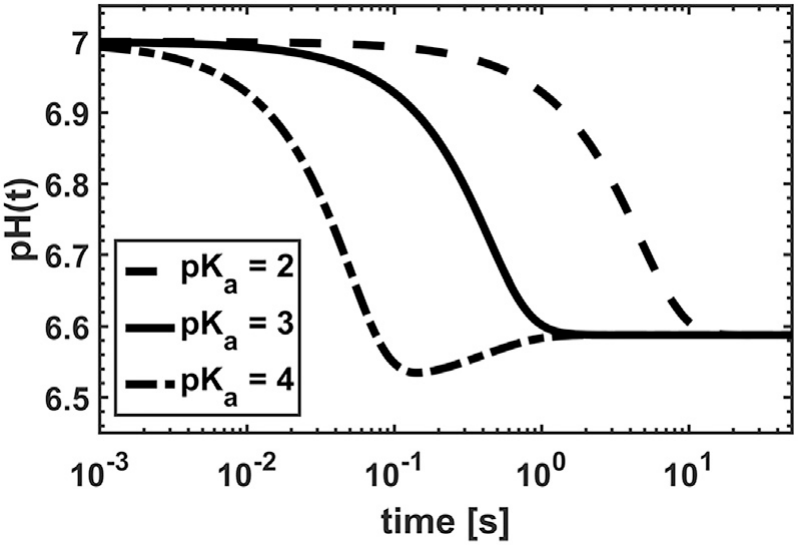
Simulated curves for a permeable weak acid. The time evolution of internal pH upon variation of the weak acid *pK*_*a*_ is shown. The following parameters were used for calculations: *pK*_1_ = 7.21, *pK*_2_ = 4, *M*_1_ = 18 cm^3^/mol, pH_0_ = ${\text{pH}}_{0}^{*}$ = 7.0, [KPi] = 100 mM, [Kpi]^∗^ = 100 mM, [calcein] = 0 M, *k*_1_ = 10^6^ s^−1^, *K*_*SV*_ = 10^2^ M^−1^, $c_{6}^{*}+c_{7}^{*}$ = 60 mM, *r*_0_ = 100 nm, and *P*_1_ = 0.003 cm/s. The parameter *pK*_*a*_ was varied as indicated in the legend.

The parameter *λ*_6_ is proportional to 1) the ratio $\left(P_{6}/P_{1}\right)$ between the acid/water permeability and 2) the ratio $\left(c_{6}^{*}/c_{s}^{*2}\right)$ between the external AH concentration $c_{6}^{*}$ and the squared solute concentration $c_{s}^{*}$. In Fig. 3, we show the effect of the permeability ratio $\left(P_{6}/P_{1}\right)$ on the dynamics of the system by fixing *P*_1_ and varying *P*_6_. The same behavior is expected if *λ*_6_ is modified upon variation of the ratio $\left(c_{6}^{*}/c_{s}^{*2}\right)$. To test this hypothesis, we fix the acid concentration at 60 mM and modify the *pK*_*a*_ of the acid (see Fig. 4). Thus, the external solute concentration is constant ($c_{s}^{*}$ = 160 mM), whereas the relative amount of the permeable species (AH $=c_{6}^{*}$) changes according to the Henderson-Hasselbach equation. Indeed, for higher *pK*_*a*_ (that is, for weaker acids), the concentration of AH gets higher with respect to the concentration of A^−^ and vice versa. As predicted, variation of the acid strength induces transitions between the three dynamic regimes similar to the permeability ratio, as shown by the similarity between Figs. 3 *c* and 4. Interestingly, the vesicle radius has no effect on the dynamic character of the system (data not shown), but only affects the relaxation rates, similarly to Fig. 2 *c*.

### Ensemble of vesicles

To describe the properties of a vesicle population as measured during osmotic-upshift relaxation experiments, we focus on ensemble-average physical quantities. To this end, we consider a polydisperse population of spherical vesicles represented by the normalized vesicle size distribution *g*(*r*_0_), where *r*_0_ is the vesicle radius. The relaxation dynamics of a single vesicle is described by the mathematical solutions of Eq. 10. These solutions depend on the surface area *A*(*r*_0_) and on the starting volume *V*_0_(*r*_0_) of the vesicle. Consequently, the dynamics of internal concentrations and volume depend on the vesicle radius, that is, *c*_*i*_ = *c*_*i*_(*r*_0_) and *V* = *V*(*r*_0_). The same holds for any physical quantity *h* function of *V* and *c*_*i*_. Thus, the population-averaged quantities $\bar{h}$ are calculated according to

(25) $$\bar{h}=\int_{0}^{\infty }h\left(r_{0}\right)g\left(r_{0}\right)\;dr_{0}.$$

**Figure 5:**
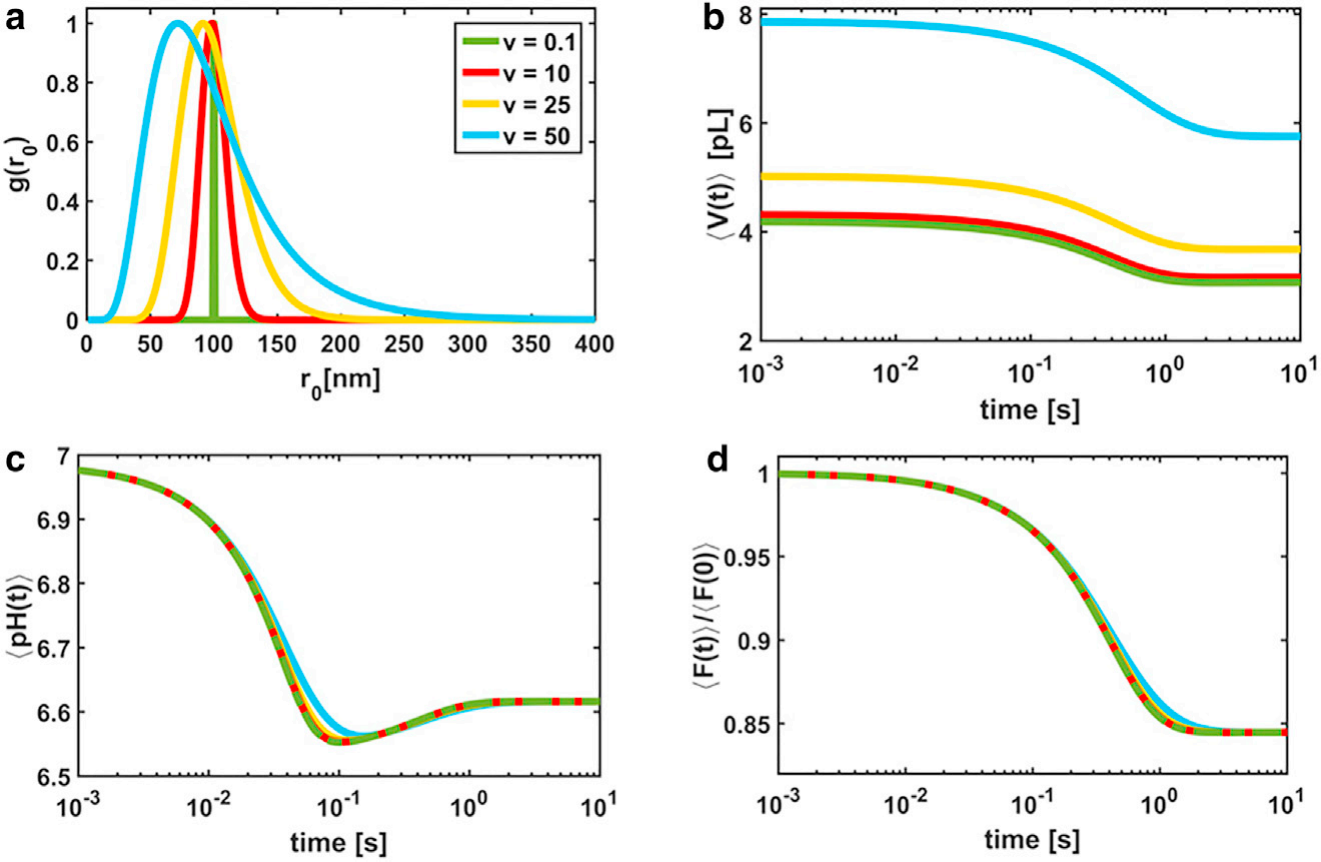
Simulated curves for different vesicle size distributions *g*(*r*_0_) and a permeable weak acid. The color code is the same in the four panels. (*a*) Log-normal distributions with mean *m* = 100 nm are shown. The variance *v* varies as indicated in the legend. (*b*) Average volume dynamics 〈V(t)〉 calculated according to Eq. 25 are shown. The average pH dynamics 〈pH(t)〉 (*c*) and the normalized average fluorescence intensity 〈F(t)〉/〈F(0)〉 (*d*) were calculated as described in Appendix B. The following parameters were used for calculations: *pK*_1_ = 7.21, *pK*_2_ = 4, *M*_*w*_ = 18 cm^3^/mol, pH_0_ = ${\text{pH}}_{0}^{*}$ = 7.0, [KPi] = 90 mM, [KPi]^∗^ = 100 mM, [calcein] = 10 mM, *k*_1_ = 10^6^ s^−1^, *K*_*SV*_ = 10^2^ M^−1^, $c_{6}^{*}+c_{7}^{*}$ = 60 mM, *r*_0_ = 100 nm, *P*_1_ = 0.003, and *P*_6_ = 0.03 cm/s. For calculation of pH(*t*), we set [KPi] = 100 mM and [calcein] = 0 M. To see this figure in color, go online.

The effects of polydispersity on the vesicle relaxation dynamics are shown in Fig. 5 for a permeable weak acid. Here, we simulated ensembles of vesicles having log-normal size distributions *g*(*r*_0_) with fixed mean (that is, *m* = 100 nm) and variable variances *ν* (see *legend* of Fig. 5 *a*). Indeed, the log-normal distribution is a good approximation of the vesicle size distribution (9,20, 21, 22). We observe that the increase of the vesicle polydispersity provokes 1) the increase of the average vesicle volume (the *curve shift* upwards in Fig. 5 *b*), 2) stretching of the relaxation curves or spread of the relaxation rates (Fig. 5 *c*), and 3) the decrease of the volume relaxation rate (Fig. 5 *d*).

## Conclusions

We present a physiochemical model to describe of the dynamics of vesicles and yeast cells upon osmotic upshift. Analysis of the computed relaxation kinetics upon osmotic upshift with an impermeable solute and a permeable weak acid allows us to draw important lessons about the system dynamics. First, the dynamic character of the relaxation kinetics depends on the number of permeable species: the number of dynamic types (or curve shapes) increases with the number of permeable molecules. Second, transitions between the different dynamic regimes are governed by the relative magnitude of the permeability coefficients of the acid and water, as well as by the ratio between the external concentration of acid and the total external solute concentration. Thus, variation of the acid strength—that is, the acid *pK*_*a*_—affects the dynamic behavior of the system. Interestingly, the dynamic behavior of the system is completely independent of the vesicle size, which affects only the relaxation rate by shifting the kinetic curve as a whole without modifying the curve shape. An important issue to consider in treating experimental relaxation data is the heterogeneity of size of the vesicle populations. Indeed, the simulated data show a stretching of the relaxation curves upon an increase of the distribution width. Therefore, in the analysis of the osmotic-upshift relaxation experiments presented in the accompanying study (4), we used vesicle size distributions measured with dynamic light scattering to account for vesicle size heterogeneities. In conclusion, the generality and flexibility of the model make it a very useful tool for the interpretation of (relaxation) kinetic experiments and for simulation of (bio)chemical and biological systems in which molecular transport across the vesicle membrane and osmoregulation play a role. While our work was under review, Hannesschlaeger et al. (23) published a mathematical model for weak acid transport across membranes that accounts both for the accompanying water flux and the presence of buffer. To the best of our understanding, this model fails to fully capture the complex interplay between volume dynamics, passive diffusion across the membrane, and reaction kinetics because the volume term, that is, $-\left(c_{i}/V\right)\dot{V}$, is missing (compare Eq. 23 in our work with (10), (11), (12), (13), (14), (15), (16) in (24)). The volume term is readily acknowledged if dynamic equations are derived from the definition of molar concentration (see Vesicle Relaxation Dynamics). We stress the importance of the volume term that couples all the equations of the system (see Eqs. 21 and 23).

## Appendix A: Derivation Of The Dimensionless Equations

To derive dimensionless equations, we express the variables appearing in Eq. 10 as a function of the dimensionless variables defined in Eq. 11, that is,

(26) $$c_{i}={\bar{c}}_{i}c_{s}^{*},\;V=\bar{V}V_{0},\;t=\bar{t}t_{c}.$$

Afterwards, these variables are substituted in Eq. 10. Upon substitution, the derivatives ${\dot{c}}_{i}$ and $\dot{V}$ become

(27) $${\dot{c}}_{i}=\frac{c_{s}^{*}}{t_{c}}\frac{\partial {\bar{c}}_{i}}{\partial \bar{t}},\;\dot{V}=\frac{V_{0}}{t_{c}}\frac{\partial \bar{V}}{\partial \bar{t}}$$

where $\left(\partial \bar{V}/\partial \bar{t}\right)=\dot{\bar{V}}$ and $\left(\partial {\bar{c}}_{i}/\partial \bar{t}\right)={\dot{\bar{c}}}_{i}$. Thus, the overall system of differential equation is

(28) $$\begin{array}{l} {\dot{\bar{c}}}_{i}=\frac{t_{c}}{\bar{V}}\left[\alpha_{wj}{\bar{c}}_{i}\left(1-\sum_{i}^{n-1}{\bar{c}}_{i}\right)+\beta_{ij}\left(\gamma_{i}-{\bar{c}}_{i}\right)\right]+\frac{t_{c}}{c_{s}^{*}}R_{ij}\\ \dot{\bar{V}}≃-t_{c}\alpha_{wj}\left(1-\sum_{i}^{n-1}{\bar{c}}_{i}\right) \end{array}$$

where the parameters $\alpha_{wj}=\frac{A}{V_{0}}P_{wj}M_{w}c_{s}^{*}$ $\left[s^{-1}\right]$, $\beta_{ij}=\left(A/V_{0}\right)P_{ij}$ [s^−1^], and $\gamma_{i}=\left(c_{i}^{*}/c_{s}^{*}\right)$ were defined. To obtain the equations in their final form as shown in the main text (Eq. 12), we set *t*_*c*_ = $\alpha_{wj}^{-1}$ and define a new dimensionless parameter $\lambda_{ij}=\left(\beta_{ij}\gamma_{i}/\alpha_{wj}\right)$ and the dimensionless reaction term ${\bar{R}}_{ij}=\left(R_{ij}/\alpha_{wj}c_{s}^{*}\right)$. For the weak acid example discussed in the main text, the dimensionless equations are the following:

(29) $$\begin{array}{l} {\dot{\bar{c}}}_{1}=0\\ {\dot{\bar{c}}}_{2}=\frac{1}{\bar{V}}{\bar{c}}_{2}\overset{˙}{\bar{V}}\\ {\dot{\bar{c}}}_{3}=\frac{1}{\bar{V}}{\bar{c}}_{3}\overset{˙}{\bar{V}}-\Omega_{1}\left({\bar{c}}_{3}-\frac{{\bar{c}}_{4}{\bar{c}}_{5}}{\Psi_{1}}\right)\\ {\dot{\bar{c}}}_{4}=\frac{1}{\bar{V}}{\bar{c}}_{4}\overset{˙}{\bar{V}}+\Omega_{1}\left({\bar{c}}_{3}-\frac{{\bar{c}}_{4}{\bar{c}}_{5}}{\Psi_{1}}\right)\\ {\dot{\bar{c}}}_{5}=\frac{1}{\bar{V}}{\bar{c}}_{5}\overset{˙}{\bar{V}}+\Omega_{1}\left({\bar{c}}_{3}-\frac{{\bar{c}}_{4}{\bar{c}}_{5}}{\Psi_{1}}\right)+\Omega_{2}\left({\bar{c}}_{6}-\frac{{\bar{c}}_{7}{\bar{c}}_{5}}{\Psi_{2}}\right)\\ {\dot{\bar{c}}}_{6}=\frac{1}{\bar{V}}{\bar{c}}_{6}\overset{˙}{\bar{V}}-\Omega_{2}\left({\bar{c}}_{6}-\frac{{\bar{c}}_{7}{\bar{c}}_{5}}{\Psi_{2}}\right)+\lambda_{6}\left(1-\frac{{\bar{c}}_{6}}{\gamma_{6}}\right)\\ {\dot{\bar{c}}}_{7}=\frac{1}{\bar{V}}{\bar{c}}_{7}\overset{˙}{\bar{V}}+\Omega_{2}\left({\bar{c}}_{6}-\frac{{\bar{c}}_{7}{\bar{c}}_{5}}{\Psi_{2}}\right)\\ \dot{\bar{V}}={\bar{c}}_{2}+{\bar{c}}_{3}+{\bar{c}}_{4}+{\bar{c}}_{5}+{\bar{c}}_{6}+{\bar{c}}_{7}-1 \end{array}$$

where the following dimensionless parameters were defined considering spherical vesicles for which $\left(A/V_{0}\right)=\left(3/r_{0}\right)$: $\Omega_{1,2}=\left(k_{1,2}/\alpha_{1}\right)=\left(r_{0}k_{1,2}/3P_{1}M_{1}c_{s}^{*}\right)$, $\Psi_{1,2}=\left(K_{1,2}/c_{s}^{*}\right)$, $\lambda_{6}=\left(P_{6}/P_{1}\right)\left(c_{6}^{*}/M_{1}c_{s}^{*2}\right)$, and $\gamma_{6}=\left(c_{6}^{*}/c_{s}^{*}\right)$.

## Appendix B: Calculation Of Probe Readout

To compare the calculated with the experimental relaxation curves, we consider the readout mechanism of the probes employed in our in vitro and in vivo assays, which are pyranine (25,26), calcein (17), and pHluorin (24).

### pH-sensitive probe readout

Pyranine and pHluorin are ratiometric pH sensors for which the ratio *R*_12_ = *F*_1_/*F*_2_ between the fluorescence intensities emitted upon excitation at two different wavelengths (453/405 nm for pyranine and 390/470 nm for pHluorin) is measured as a function of known pH-values during calibration experiments. Afterwards, unknown pH-values are calculated from the measured fluorescence ratios *R*_12_ by using the empirical calibration curve pH(*R*_12_)_*exp*_. Here, we perform the opposite procedure; that is, we calculate the population-averaged mean ratio ${\langle R_{12}\left(t\right)\rangle }_{th}$ from the computed pH relaxation curves pH(*r*_0_, *t*) and compare it with the experimentally determined ratio ${\langle R_{12}\left(t\right)\rangle }_{exp}$. To this end, we must consider the fluorescence signal generated by an ensemble of vesicles containing the probes at concentration *c*_0_ and with size distribution *g*(*r*_0_). The fluorescence intensity *F* [photon/s] emitted by a single vesicle is

(30) F=fN

where *N* is the number fluorophore in the vesicle lumen. For the pH sensor, the molecular brightness *f* [photon/(s ⋅ molecule)] is sensitive to the pH in solution, that is, *f* = *f*(pH). Thus, the intensity *F* is also a function of the pH, that is, *F* = *F*(pH). For a vesicle population, the number of fluorophores *N* per vesicle is distributed according to the Poisson distribution *P*_*N*_,

(31) $$P_{N}\left(r_{0}\right)=\frac{\lambda {\left(r_{0}\right)}^{N}e^{-\lambda \left(r_{0}\right)}}{N!}$$

with mean *λ*(*r*_0_) = *c*_0_*V*(*r*_0_)*N*_*a*_ (27,28), where *V*(*r*_0_) = $\left(4/3\right)\pi r_{0}^{3}$ is the vesicle volume of a spherical vesicle. Accordingly, the mean intensity $\langle F\left(r_{0}\right)\rangle$ emitted by an ensemble of vesicle with radius *r*_0_—that is, a monodisperse population for which *g*(*r*_0_) = *δ*(*r* − *r*_0_)—is simply $\langle F\left(r_{0}\right)\rangle =f\lambda \left(r_{0}\right)$. Instead, for a polydisperse population of spherical vesicles, the mean intensity 〈F〉 is

(32) $$\langle F\rangle =\frac{4}{3}\pi c_{0}N_{a}\int_{0}^{\infty }f\left(pH\right)\;r_{0}^{3}\;g\left(r_{0}\right)\;dr_{0}.$$

Importantly, we note that 〈F〉 scales with $r_{0}^{3}$, which means that larger vesicles contribute more to the measured signal than smaller ones. Next, we calculate the average fluorescence ratio ${\langle R_{12}\rangle }_{th}$ between the mean intensities $\langle F_{1}\rangle$ and $\langle F_{2}\rangle$, that is,

(33) $${\langle R_{12}\rangle }_{th}=\frac{\langle F_{1}\rangle }{\langle F_{2}\rangle }=\frac{\int_{0}^{\infty }f_{1}\left(pH\right)\;r_{0}^{3}\;g\left(r_{0}\right)\;dr_{0}}{\int_{0}^{\infty }f_{2}\left(pH\right)\;r_{0}^{3}\;g\left(r_{0}\right)\;dr_{0}}.$$

Here, we recall that the pH dynamics are a function of the vesicle radius, that is, pH = pH(*r*_0_, *t*). Therefore, the molecular brightness *f*(pH) is also a function of *r*_0_ and cannot be extracted from the integral in Eq. 33. The theoretical relationship *f*(pH) between the molecular brightness and the pH is given for pyranine in (29). However, for the sake of simplicity, we derive a semiempirical equation exploiting the calibration data of the pH sensor for the calculation of ${\langle R_{12}\rangle }_{th}$. To this end, we rewrite Eq. 33 upon multiplication by the ratio $\left(f_{1}\left(pH_{0}\right)/f_{1}\left(pH_{0}\right)\right)\left(f_{2}\left(pH_{0}\right)/f_{2}\left(pH_{0}\right)\right)$ as follows:

(34) $${\langle R_{12}\rangle }_{th}=R_{12}\left(pH_{0}\right)\frac{\int_{0}^{\infty }R_{1}\left(pH\right)\;r_{0}^{3}\;g\left(r_{0}\right)\;dr_{0}}{\int_{0}^{\infty }R_{2}\left(pH\right)\;r_{0}^{3}\;g\left(r_{0}\right)\;dr_{0}}$$

where the constant *R*_12_(pH_0_) and the functions *R*_1_(pH) and *R*_2_(pH) are

(35) $$\begin{array}{l} R_{12}\left(pH_{0}\right)=\frac{f_{1}\left(pH_{0}\right)}{f_{2}\left(pH_{0}\right)}=\frac{F_{1}\left(pH_{0}\right)}{F_{2}\left(pH_{0}\right)}\\ R_{1}\left(pH\right)=\frac{f_{1}\left(pH\right)}{f_{1}\left(pH_{0}\right)}=\frac{F_{1}\left(pH\right)}{F_{1}\left(pH_{0}\right)}\\ R_{2}\left(pH\right)=\frac{f_{2}\left(pH\right)}{f_{2}\left(pH_{0}\right)}=\frac{F_{2}\left(pH\right)}{F_{2}\left(pH_{0}\right)} \end{array}$$

upon normalization with respect to the brightness at pH_0_ = 7. These functions are obtained from empirical fits of the calibration data, which are {*F*_1_, *F*_2_, pH}. Finally, by numerical integration of Eq. 34, we can calculate the pH sensor readout as a function of time ${\langle R_{12}\left(t\right)\rangle }_{th}$ from the computed pH relaxation curves pH(*r*_0_, *t*) and the vesicle size distribution *g*(*r*_0_). This allows us to compare the calculated ${\langle R_{12}\left(t\right)\rangle }_{th}$ with the measured ${\langle R_{12}\left(t\right)\rangle }_{exp}$ relaxation kinetics. Also, we can use the empirical function pH(*R*_12_) to obtain ${\langle pH\left(t\right)\rangle }_{th}$. For pyranine, we used the following empirical functions for the calculation of ${\langle pH\left(t\right)\rangle }_{th}$:

1) *R*_12_(pH_0_) = 0.5133;
2) *R*_1_(pH) = 0.5129 × pH^2^ − 5.793 × pH + 16.4;
3) *R*_2_(pH) = $a_{1}e^{b_{1}pH}+c_{1}e^{d_{1}pH}$ with *a*_1_ = 1325, *b*_1_ = −0.9138, *c*_1_ = −9111, and *d*_1_ = −1.278;
4) pH(*R*_12_) = $a_{2}e^{b_{2}R_{12}}+c_{2}e^{d_{2}R_{12}}$ with *a*_2_ = 6.643, *b*_2_ = 0.1072, *c*_2_ = −0.9937, and *d*_2_ = −8.149.

### Calcein readout

To calculate the readout of calcein, we follow a similar approach as for the pH sensor, but instead of the ratio ${\langle R_{12}\left(t\right)\rangle }_{th}$, we calculate the normalized ratio ${\langle F_{r}\left(t\right)\rangle }_{th}$, that is,

(36) $${\langle F_{r}\left(t\right)\rangle }_{th}=\frac{\langle F\left(t\right)\rangle }{\langle F\left(0\right)\rangle }=\frac{\int_{0}^{\infty }f\left(c\left(t\right)\right)\;r_{0}^{3}\;g\left(r_{0}\right)\;dr_{0}}{\int_{0}^{\infty }f\left(c\left(0\right)\right)\;r_{0}^{3}\;g\left(r_{0}\right)\;dr_{0}}.$$

Indeed, the molecular brightness of calcein at self-quenching concentration is a function of the calcein concentration itself, that is, *f* = *f*(*c*). Because, in our description, the calcein concentration at time 0 *c*(0) is independent from *r*_0_, the term *f*(*c*(0)) is constant. Thus, we can take this term outside the integral and write

(37) $${\langle F_{r}\left(t\right)\rangle }_{th}=\frac{\int_{0}^{\infty }F_{r}\left(r_{0},t\right)\;r_{0}^{3}\;g\left(r_{0}\right)\;dr_{0}}{\langle r_{0}^{3}\rangle }$$

where $\langle r^{3}\rangle =\int r_{0}^{3}g\left(r_{0}\right)\;dr_{0}$ is the average cubic radius and *F*_*r*_(*r*_0_, *t*) is a function of the internal concentration *c*(*r*_0_, *t*) according to the Stern-Volmer equation

(38) $$F_{r}\left(r_{0},t\right)=\frac{F\left(r_{0},t\right)}{F\left(0\right)}=\frac{f\left(c\left(t\right)\right)}{f\left(c\left(0\right)\right)}=\frac{1+K_{SV}c\left(0\right)}{1+K_{SV}c\left(r_{0},t\right)}.$$

Equation 37 is used to calculate the calcein readout ${\langle F_{r}\left(t\right)\rangle }_{th}$ from the dynamic quenching constant *K*_*SV*_, the vesicle size distribution *g*(*r*_0_), the starting calcein concentration *c*(0), and the computed relaxation curves *c*(*r*_0_, *t*).
